# Prevalence of Antibodies against Hantaviruses in Serum and Saliva of Adults Living or Working on Farms in Yorkshire, United Kingdom

**DOI:** 10.3390/v6020524

**Published:** 2014-02-05

**Authors:** Lisa J. Jameson, Autilia Newton, Louise Coole, Edmund N. C. Newman, Miles W. Carroll, Nick J. Beeching, Roger Hewson, Robert M. Christley

**Affiliations:** 1 Microbiology Services, Public Health England, Porton Down, Wiltshire, SP4 0JG, UK; E-Mails: edmund.newman@phe.gov.uk (E.N.C.N.); miles.carroll@phe.gov.uk (M.W.C.); roger.hewson@phe.gov.uk (R.H.); 2 Institute of Infection and Global Health, University of Liverpool, Liverpool, L69 7BE, UK; E-Mail: robc@liverpool.ac.uk; 3 Public Health England, Yorkshire and the Humber, York, YO41 1LZ, UK; E-Mail: autilia.newton@phe.gov.uk; 4 Field Epidemiology Services, Public Health England, Leeds, LS2 7UE, UK; E-Mail: louise.coole@phe.gov.uk; 5 Clinical Sciences, Liverpool School of Tropical Medicine, Liverpool, L3 5QA, UK; E-Mail: beeching@liverpool.ac.uk

**Keywords:** farmers, hantavirus, serology, saliva, serosurveillance, haemorrhagic fever with renal syndrome, Seoul virus

## Abstract

Hantaviruses are an established cause of haemorrhagic fever with renal syndrome (HFRS) in Europe. Following a confirmed case of HFRS in the UK, in an individual residing on a farm in North Yorkshire and the Humber, a tidal estuary on the east coast of Northern England, and the subsequent isolation of a Seoul hantavirus from rats trapped on the patient’s farm, it was considered appropriate to further investigate the public health risk of this virus in the region. Of a total 119 individuals tested, nine (7.6%) were seropositive for hantavirus antibodies. Seven of the seropositive samples showed a stronger reaction to Seoul and Hantaan compared to other clinically relevant hantaviruses. Observation of rodents during the day, in particular mice, was associated with a reduced risk of seropositivity. In addition to one region known to be at risk following an acute case, five further potential risk areas have been identified. This study supports recently published evidence that hantaviruses are likely to be of public health interest in the region.

## 1. Introduction

Hantaviruses (genus *Hantavirus*, family *Bunyaviridae*) are a globally distributed group of emerging rodent- and insectivore-borne RNA viruses named after the prototype strain, Hantaan virus (HTNV) discovered in Asia. Several distinct species are known to circulate; those confirmed to cause human disease within Europe are: Dobrava (DOBV), Puumala (PUUV), Tula (TULV), Saaremaa (SAAV) and Seoul (SEOV) viruses [1]. Andes (ANDV) and Sin Nombre (SNV) are the predominant hantavirus species responsible for serious human disease in South and North America, respectively [2].

Each hantavirus has a specific rodent or insectivore species acting as its natural reservoir which intermittently excretes infectious virus in urine, saliva and faeces [3,4]. Seoul is a unique member of the hantavirus family in that it is considered to have a potential for global distribution due to its reservoir host, the brown rat (*Rattus norvegicus*) being ubiquitous on all continents with the exception of Antarctica. Viral transmission to humans typically occurs when materials contaminated with rat excreta are disturbed causing virus particles to aerosolise and be inhaled. Contact between broken mucosal membranes and virus contaminated materials or through a direct bite are also potential routes of transmission [1].

Known risk populations for contracting haemorrhagic fever with renal syndrome (HFRS) are rural workers, military personnel, underground workers and pest controllers; as their occupation brings them in close contact with rodents [2,5]. Severe disease is more frequently detected in persons between the ages 20–50 years old. The disease has been reported in both sexes; however, possibly linked to occupational risk there is a significantly higher prevalence in males [1,3]. 

The most common and convenient method for determining exposure to hantaviruses is detection of specific antibodies [6]. In the United Kingdom (UK) several studies have used this method for determining seroprevalence of hantavirus immunoglobin G (IgG) in apparently healthy persons. One assessment of serostatus in Northern Ireland found 1.2% (4/320) of farmers had antibodies against hantavirus [7]. The most recent serosurvey of 606 farmers, farm workers and their families in Herefordshire and Lancashire, England, demonstrated a seroprevalence of 4.7% in the first year and 4.8% in the second year of the study [8]. Both studies have provided evidence for hantavirus exposure in the UK; however, it was not until 2012 that a causative hantavirus species was confirmed. Rodent trapping on the farm of an acutely ill patient led to the identification and isolation of the first UK strain of hantavirus from wild rats (*Rattus norvegicus*); a SEOV designated Humber [9]. The patient was a resident on a small livestock farm in North Yorkshire and Humber. The patient disclosed regular exposure to rats with a noticeable increase in rat numbers in the months preceding their illness. Two of four rats trapped and tested from the farm were positive for SEOV RNA. 

With a confirmed autochthonous case of HFRS and virus isolation from the local rat population, further investigations were undertaken to determine the extent of human exposure to this virus in the region. The primary objective of the present study was to determine the seroprevalence of hantavirus antibodies in an occupationally exposed group in a known risk area of the UK. 

A further objective was to identify a more convenient method of sampling for future studies. The use of saliva is an increasing area of research for diagnostic sampling as it is easy to collect, non invasive to the patient and readily available with healthy adults producing between 500–1,500 mL of saliva per day [10]. Whilst the predominant immunoglobulin isotype in saliva is immunoglobin A (IgA), IgG is also active within the oral cavity where it is mainly derived from gingival cervicular fluid and mucosal transduate [10,11]. A correlation between saliva IgG and serum IgG has been demonstrated for several viruses including human immunodeficiency virus, hepatitis A virus, hepatitis C virus, Epstein Barr virus, cytomegalovirus and rubella virus [10]. To the best of our knowledge, no published survey has researched the use of saliva collection for detection of IgG levels specific to hantaviruses.

## 2. Results

128 volunteers were recruited but nine were excluded from analysis because of ineligibility. Nine (7.6%; 95% CI 4.0 to 13.8%) of the 119 eligible volunteers had detectable levels of IgG serum antibodies against hantaviruses. As demonstrated in Table 1, 7/9 positive sera reacted strongly to HTNV and/or SEOV and 2/9 positive sera reacted strongly to SNV and/or PUUV. 112/119 disclosed some form of travel outside of the UK in their lifetime; crucially the two volunteers whose serum cross-reacted with SNV had not travelled outside of Europe. From the 119 eligible volunteers, 117 also supplied a saliva sample. From the serum positive individuals 8/8 corresponding saliva samples showed characteristic positive fluorescence compared to negative saliva samples (13/13) tested. One saliva sample was not collected from a volunteer with positive serum and therefore was unable to be tested.

**Table 1:** Pattern of reactivity for immunofluorescence assay at 1:100 dilution in sera and (saliva).

Sample	DOBV	HTNV	PUUV	SAAV	SNV	SEOV	Result
1	− (−)	− (+)	++ (+)	− (−)	++ (+)	+ (++)	PUUV/SNV
2	− (−)	++ (+)	− (+)	+ (+)	− (+)	++ (+)	HTNV/SEOV
3	− (−)	+ (+)	+ (+)	− (−)	+ (+)	++ (+)	SEOV
4	− (−)	− (+)	+++ (−)	− (−)	− (+)	+ (+)	PUUV
5	− (+)	+++ (+)	− (−)	− (−)	− (+)	++ (+)	HTNV/SEOV
6	− (+)	++ (+)	− (−)	− (−)	− (+)	+ (+)	HTNV/SEOV
7	− (−)	+++ (++)	− (+)	− (−)	− (+)	++ (++)	HTNV/SEOV
8	−	++	−	−	−	+	HTNV/SEOV
9	− (+)	++ (++)	− (+)	− (+)	− (+)	+ (++)	HTNV/SEOV

Reactivity score: − negative, + weak, ++ moderate, +++ strong.

**Figure 1:**
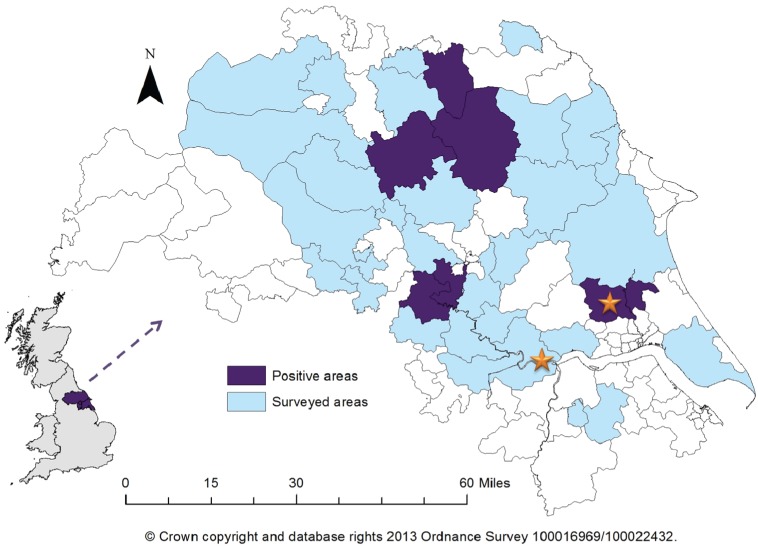
Map of Yorkshire and the Humber showing surveyed areas and areas with a minimum of one positive serum sample recorded. Stars indicate location of previous acute cases. Insert shows location of Yorkshire within the United Kingdom.

The majority of volunteers (98/119) disclosed their main or full-time occupation to be farming; the remaining volunteers (21/119) reside on a farm. Postcode information was collected for 115/119 eligible participants allowing geographical mapping of seropositive individuals; 2/4 volunteers who did not provide this information were seropositive, with both samples identifying exposure to PUUV/SNV-like hantavirus. 6/34 districts included in the survey had at least one positive sample (Figure 1). One of the six regions was previously known to be a risk area due to one of the acute cases occurring there, however five did not have any previous information on hantavirus prevalence.

As detailed in Table 2, analyses from the questionnaire results showed little association between any measured demographic or farm specific variables and serostatus. In contrast, differences in rodent contact identified potential areas for future investigation. All seropositive individuals confirmed they had rodents on their property with 9/9 reporting rats and 6/9 mice. Seeing rodents during the day appears to give a significant reduction in risk (p > 0.001, OR 0.006) with only 2/9 positive individuals noticing rodents during the day compared to 9/9 seeing rodents at night. Further investigation of which rodent species are seen highlighted that reporting mice on the farm was also associated with decreased risk (p= 0.02, OR 0.1, 95% CI 0.02 to 0.96). 

**Table 2:** Analysis of factors associated with seropositivity to hantaviruses.

Category	No. positive (%)	No. Negative	No. tested	Odds ratio	CI	p-value*
*Age (years)*						
<30	3 (14.3)	18	21	2.5	0.4 to 13.2	0.2
≥30	6 (6.1)	92	98			
Median	45	51				
*Gender*						
Male	7 (7.0)	93	100			
Female	2 (10.5)	17	19	1.6	0.1 to 9.2	0.6
*Farmer*						
Yes	8 (8.2)	90	98	1.8	0.2 to 82.7	>0.99
No	1 (4.8)	20	21			
*Farm type*						
Animal	4 (11.4)	31	35			0.7
Arable	1 (7.1)	13	14	1.7	0.1 to 88.8	
Mixed	4 (5.7)	66	70	1.9	0.3 to 11	
*Farm classification*						
Cereal	5 (6.3)	75	80	0.7	0.1 to 3.9	0.7
Cropping	3 (7.5)	37	40	1.1	0.2 to 5.5	>0.99
Horticulture	0	4	4	NA		
Pig	1 (5.9)	16	17	0.8	0.02 to 6.7	>0.99
Poultry	3 (15.0)	17	20	3.0	0.4 to 15.6	0.1
Dairy	1 (5.6)	17	18	0.8	0.02 to 6.2	>0.99
Livestock	7 (8.1)	79	86	1.8	0.3 to 18.2	0.7
Other	0	3	3	NA		
*Farm size*						
<500	6 (7.2)	77	83			
≥500	3 (9.4)	29	32	2.0	0.5 to 11.5	0.4
Median	330	296				
*Materials*						
Silage	9 (9.8)	83	92	NA	0.8 to Inf	0.06
Bedding	9 (8.6)	96	105	NA	0.4 to Inf	0.4
Feed	9 (9.1)	90	99	NA	0.6 to Inf	0.2
Hay	8 (9.3)	78	86	4.2	0.5 to 190.1	0.3
Timber	7 (6.7)	97	104	0.8	0.1 to 8.4	0.7
Coal	6 (9.8)	55	61	2.3	0.5 to 15.0	0.3
*Rodents seen*						
**Mice**	**6 **(**5.1**)	**112**	**118**	**0.1**	**0.02** to **0.96**	**0.02**
Rats	9 (7.6)	109	118	NA	0.2 to Inf	>0.99
**During day**	**2 **(**1.7**)	**117**	**119**	**0.006**	**0.0004** to **0.05**	<**0.001**
At night	9 (7.6)	109	118	NA	0.2 to Inf	>0.99
*Rodent control*						
Professional	4 (14.3)	24	28	2.8	0.5 to 14.2	0.2
Self	5 (5.6)	85	90			

* Calculated using Fischer’s exact test.

## 3. Discussion

In the UK, there is a lack of contemporary data available on the prevalence of hantavirus antibodies in at-risk populations. Only isolated cases have been reported with the majority of seroprevalence studies undertaken in the 1980s and 1990s. Uncertainty surrounding the presence of a hantavirus in the UK has been resolved following a recent cases of acute hantavirus infection in North Yorkshire and the Humber region which led to the characterisation and isolation of a UK variant of SEOV from wild *R. norvegicus*. As brown rats are the most likely known source for hantavirus infections in the region, farmers and those who reside on a farm were chosen for this study as they have an increased risk for contact with rats and their excrement. While it was expected contact between the study population and rats would be higher than the general population it was surprising to find 92.4% of questionnaire respondents regularly seeing rats on their residence. From a public health perspective this is of concern given that SEOV is not the only pathogen of concern transmissible from rats as previously reviewed by Webster [12]. 

The most comparable study, over 15 years ago, looking at exposure to a variety of zoonotic organisms in English farmers found seroprevalence to hantaviruses to be 4.7% [8]. Although our current study found the frequency to be slightly higher at 7.6%, the previous estimate lies within our 95% confidence interval. Nevertheless, the current study may be expected to identify a higher prevalence as the study area included a known risk area whereas the previous study’s locations had no link to symptomatic hantavirus infection. 

We found no significant association between contact with different agricultural materials and hantavirus seroprevalence. Neither did we find any significant association between hantavirus infections and various demographic factors such as age or gender. This may be a direct result of the low number of positives, low uptake of women and older volunteers and the study population encompassing a convenience rather than random sampling strategy. Due to the low number of total positive volunteers the analysis of individual factors such as gender and farm type was limited and therefore may inaccurately imply there is no correlation. In retrospect it would have been valuable to have sampled more volunteers under the age of 30. This may have provided some indication as to whether SEOV was introduced recently to the region or has been circulating for some time.

Saliva samples from 8/9 (1 sample not collected) volunteers with positive serum samples demonstrated reactive IgG antibodies against hantaviruses. No other alterations were made to the assay other than sample type. Whilst saliva appears to be promising for determining a simple positive sample it appears to be less suitable for identifying serotype. Most samples showed a decrease in recorded fluorescence in comparison to serum samples and were less specific with at least mild reactivity recorded for SNV, SEOV and HTNV for all eight samples tested. A further weakness of the use of saliva was discovered following repeated testing of the samples up to 11 months after collection; samples which originally demonstrated reactivity no longer did so. It is most likely this is due to sample degradation as saliva contains many micro-organisms and proteases which may affect sample stability. Repeated freeze thaws and a lack of addition of additives to reduce degradation such as, sodium azide or protease inhibitors may have contributed to this. Nonetheless collection of saliva is a convenient and less-invasive sampling method for serosurveillance surveys with proven effectiveness for other viruses, and its potential use for hantavirus seroprevalence studies is worth further investigation.

It was expected that the majority of serum samples reactive to SEOV would show considerable cross-reactivity with HTNV, a known issue using this method. The finding of antibodies to hantaviruses other than SEOV in two individuals was not unexpected. An acute human case in 2010 with symptoms typical of HFRS produced a similar result with serological assays indicating cross-reaction with a PUUV/SNV-like hantavirus [13]. In addition, Ahlm *et al.* [14] reported similar findings with three samples reacting to SNV in a study of Swedish farmers. This raises the prospect of another circulating hantavirus in the UK, most likely PUUV as SNV has not been detected outside of the Americas, for which the environmental factors have already been demonstrated to be suitable and the reservoir host (*Myodes glareolus*) is ubiquitous [15]. One further possibility is a novel UK hantavirus. Recently, Pounder *et al.* [16] described detection of a vole-associated hantavirus in north-west England. Blood from the field vole (*Microtus agrestis*) demonstrated cross-reactivity with PUUV using an indirect fluorescent antibody test but molecular techniques suggested it to be distinct from other classified hantavirus species. Neutralisation is not a technique routinely used at PHE Porton for examination of hantavirus infection. However, determination of the specific hantavirus in individual samples that are reactive to would be of interest particularly for the PUUV/SNV serum samples, and this will be considered in future.

One variable of statistical significance, highlighted by this serosurvey was the presence of rodents during the day; surprisingly this appeared to be protective. An explanation for this may reside with the questionnaire design. Volunteers were asked if rodents were observed during the day, this was not further explored as to whether it was rats or mice seen during the day. In the absence of an extremely large population, rats are rarely seen during the day whereas mice are less constrained by such population dynamics. Mice are naturally averse to the presence of rats, therefore their presence may indicate a lower likelihood of rats and in turn a decreased potential exposure to SEOV. This is further supported by volunteers who reported seeing mice on their property being less likely to be seropositive. 

Future investigations should look to include analysis of risk areas for hantavirus and flood zones which was out-with the scope of this preliminary study. This is particularly prudent given that North Yorkshire and the Humber is a high risk area for floods within the UK. Flooding is a recognised trigger for outbreaks of rat-borne diseases, in particular leptospirosis but also hantavirus, due to changes in rat behaviour leading to increased contact with humans [17]. 

## 4. Materials and Methodology

### 4.1. Study Location

North Yorkshire and the Humber is located in the region of Yorkshire, North East England 53°57′30″N 1°4′49″W (York). The distribution of farm types varies across the county: livestock is predominant in the north-west, cereal and general cropping in the east/south-east, with the north, north-east and central areas generally more fragmented with a mixture of farm types.

It has a population of approximately 1, 700,000 within which there is generally an even gender ratio, with the exception of 80+ where females: male ratio is ~1.8:1. North Yorkshire has a high proportion (24%) of its population over the age of 65. The study area incorporated the following locations: Craven, East Riding of Yorkshire, Hambleton, Harrogate, Hull, North East Lincolnshire, North Lincolnshire, North Yorkshire, Richmondshire, Ryedale, Scarborough, Selby and York. 

### 4.2. Study Subjects

Subjects included in the study were adult volunteers (≥18 years old on day of sampling) who verbally confirmed they live or work on a farm within the study area and who consented to blood donation for the purpose of anonymous screening for the presence of antibodies against hantavirus. This group was selected because of their presumed increased risk of exposure to potential reservoirs and as representative of the population to which the acute case belonged.

Recruitment was undertaken between February and April 2013. A convenience sample of volunteers was obtained through local press releases, newsletters and recruitment drives at local meetings and markets. Volunteers were provided with study information and a consent form before sample collection. A questionnaire was designed and piloted following informed discussions with members of the farming community. The questionnaire was completed by face to face interview and included sections on occupation, working conditions, travel history and contact with rodents. 

Blood was collected by venepuncture and serum separated from the blood using standard methods of density gradient centrifugation [18]. Saliva was collected using the Salivette system (Sarstedt Ltd., Leicester, UK) with saliva separated as described by Lamey and Nolan [19]. Serum and saliva samples were stored frozen at −80 °C until tested. This study was approved by NHS National Research Ethics Service reference 05/Q2008/7.

### 4.3. Data Processing and Statistical Analysis

Sample size calculation was based on the most recent and comparable survey where 4.7% of farmers were seropositive [8]. A minimum sample size of 73 was calculated to be sufficient to estimate the proportion seropositive, assuming the true prevalence is 5%, with 95% confidence level and 5% precision. As sampling was planned to occur at farmer meetings and markets, the sample size was recalculated to account for clustering assuming an intra-cluster correlation coefficient of 0.1 as n1 = n(1 + p(m − 1)), where n is the estimated sample size assuming simple random sampling, n1 is the new estimate of the required sample size, p is the intra-cluster correlation coefficient and m is the number of clusters (here assumed to be 5; [20]). Hence, the revised sample size was approximately 100. 

Statistical analyses were performed with the software program Minitab version 16 (Minitab Inc., State College, PA, USA) and the R language for statistical computing [21]. Fisher’s exact test was used to compare proportions. This approach did not account for the clustering within the data (due to the selection process) and hence may result in increased risk Type I errors. However, the goal of this analysis was hypothesis generation rather than hypothesis testing and hence this limitation was accepted. The results of theses comparisons must be interpreted in light of this, and the limited statistical power of the study to detect differences in seropositivity between exposure groups. Significance was set at p < 0.05; 95% confidence intervals for proportions were calculated using the Wilson Method [22].

### 4.4. Serological Test

All sera were screened for presence of hantavirus IgG using anti-hantavirus indirect immunofluorescence test mosaic 1 (Euroimmun, Luebeck, Germany) in accordance with the manufacturer’s recommendations. Each sample was compared to a known positive (Euroimmun CI 278h-0101-1G) and a negative control (human sera). The chosen assay screens for antibodies against the most clinically relevant pathogenic hantavirus species (DOBV, HTNV, PUUV, SAAV, SEOV and SNV). Samples showing repeatable characteristic cytoplasmic fluorescence at a dilution of 1:100 were considered positive. For each positive serum sample the corresponding saliva sample was screened using the same test and conditions as that for the sera. Thirteen random saliva samples were screened as negative controls.

## 5. Conclusions

While it is expected that farmers will have higher seroprevalence rates than the general population, this study is useful in furthering the understanding of hantaviruses, and most likely SEOV, in North Yorkshire and Humber and will aid future studies with a view to reducing risk. Seoul virus has previously been considered to be mainly an urban hantavirus almost exclusively reported in Asia. However, our results support the assumption of widespread rural circulation of SEOV in the region. In addition to the recent acute clinical case, five further areas of the county demonstrate seropositivity and regular contact with a common carrier host; therefore hantavirus should be included in the differential diagnosis of patients with suspected leptospirosis in the North Yorkshire and Humber region.
